# Manual Placenta Removal is Associated with Increased Postpartum Prescriptions of Antibiotics: a Retrospective Cohort Study of Data from the Anti-Infection Tool

**DOI:** 10.1007/s13224-022-01696-x

**Published:** 2022-08-24

**Authors:** Amanda Janson, Claes Ignell, Andrea Stuart

**Affiliations:** 1 Department of Obstetrics and Gynecology, Helsingborg hospital, Helsingborg, Sweden; 2 Department of Clinical Sciences, Lund University, Malmö, Sweden; 3 Department of Clinical Sciences, Lund University, Lund, Sweden

**Keywords:** Manual placenta removal, Postpartum infection, Antibiotic prescription, Anti-Infection tool, Endometritis

## Abstract

**Purpose:**

No consensus exists whether to administer prophylactic antibiotics in conjunction with manual placenta removal. This study aimed to investigate the postpartum risk of a new prescription of antibiotic treatment, a possible indirect variable for infection, after manual placenta removal.

**Methods:**

Obstetric data were merged with data from the Anti-Infection Tool (Swedish antibiotic registry). All vaginal deliveries (*n* = 13 877) at Helsingborg Hospital, Helsingborg, Sweden, from January 1st, 2014 until June 13th, 2019 were included. Diagnosis codes for infection can be lacking, while the Anti-Infection Tool is complete as it is unavoidable in the computerized prescription system. Logistic regression analyses were performed. The risk of a prescription of antibiotics 24 h to 7 days postpartum was analyzed in the entire study population, and in a subgroup of women not having received any antibiotics 48 h prior to delivery until 24 h after delivery, referred to as “antibiotic-naïve.”

**Results:**

Manual placenta removal was associated with an increased risk of an antibiotic prescription, adjusted (a) OR = 2.9 (95%CI 1.9–4.3). In the antibiotic-naïve subgroup, manual placenta removal was associated with an increased risk of antibiotic prescription, in general, aOR = 2.2 (95%CI 1.2–4.0), endometritis-specific antibiotics, aOR = 2.7 (95%CI 1.5–4.9), and intravenous antibiotics, aOR = 4.0 (95%CI 2.0–7.9).

**Conclusion:**

Manual placenta removal is associated with an increased risk of antibiotic treatment postpartum. An antibiotic-naïve population might benefit from prophylactic antibiotics to reduce the risk of infection, and prospective studies are needed.

## Introduction

Retained placenta occurs after approximately 2% of deliveries. Risk factors include previous placenta retention, prolonged labor, preterm delivery, and multiple pregnancies [1–4]. Placenta retention is associated with a fivefold increased risk of major obstetric hemorrhage [5]. The main treatment is prompt manual removal of the placenta [1]. Whether manual placenta removal (MPR) is associated with an increased risk of endometritis is still debated [6]. Swedish national guidelines do not recommend prophylactic antibiotics to women undergoing MPR. In a retrospective cohort study with 25,687 deliveries, MPR was associated with a threefold risk of postpartum endometritis [7]. A Swedish study from 2018 concluded a similar increased risk [8]. Other studies have shown no significant correlations between MPR and postpartum endometritis [4, 9]. A majority of previous studies are based on diagnosis codes, which can be lacking [10].

The aim of the study was to assess the postpartum risk of a new prescription of antibiotics, as a indirect variable of infection and unavoidable to be registered in the computerized prescription system, in patients having undergone MPR compared with spontaneous placental delivery.

## Material and Methods

All women (*n* = 13 877) giving birth vaginally at Helsingborg Hospital from January 1st, 2014 until June 13th, 2019 were included in the study. Women with multiple gestation were only included once.

Data were merged from the medical record system (Obstetrix, Cerner Corporation, North Kansas City, Missouri, USA) and the nationwide database recording all antibiotic prescriptions (Anti-Infection Tool) [11, 12]. Registration in the Anti-Infection Tool is unavoidable in the computerized system.

Gestational age was calculated using estimated date of delivery at the first ultrasound and date of delivery.

ICD-10 procedure codes [10]: Manual placenta removal (MBA10, MBA30), epidural (DA002, SN999); blood transfusion (DR029, DR030, DR036). Diagnoses related to infection were defined using ICD-10 codes [10]: endometritis (O859); puerperal fever (O864); unspecified postpartum infection (O861, O868 and O983).

Antibiotics were classified according to the Anatomical Therapeutic Chemical (ATC) classification system [13] : Antibiotics mainly used to treat endometritis: amoxicillin and enzyme inhibitors (J01CR02), cefadroxil (J01DB05), cefotaxime (J01DD01), ceftazidime (J01DD02), ceftibuten (J01DD14), cefuroxime (J01DC02), gentamicin (J01GB03), imipenem and enzyme inhibitors (J01DH51), clindamycin (J01FF01), metronidazole (P01AB01), metronidazole (J01XD01). Intravenous antibiotics: intravenous penicillin: piperacillin and enzyme inhibitors (J01CR05), cloxacillin (J01CF02), ampicillin (J01CA01); intravenous cephalosporins: cefuroxime (J01DC02), cefotaxime (J01DD01), ceftazidime (J01DD02); benzylpenicillin (J01CE01); gentamicin (J01GB03); imipenem and enzyme inhibitors (J01DH51); clindamycin (J01FF01); intravenous metronidazole (J01XD01).

Prophylactic antibiotics in conjunction with MPR are not recommended. Postpartum endometritis is treated with a combination of cephalosporins and metronidazole, oral or intravenous. Mild cases are treated with amoxicillin and enzyme inhibitors.

The main end point of the study was a new prescription of antibiotics 24 h to 7 days postpartum. Multiple antibiotic prescriptions to one and the same patient within this time slot were recorded as one prescription. Analyses were also performed focusing on endometritis-antibiotics and intravenous antibiotics.

Fourteen potential confounding variables for postpartum antibiotic prescription were identified: Maternal age (years), body mass index (BMI, divided into class variables with increments of five), primiparity (yes/no), multiple gestation (yes/no), gestational age (weeks + days), smoking during pregnancy (yes/no), induction of labor (yes/no), epidural anesthesia (yes/no), prelabor rupture of membranes (PROM, yes/no), vacuum extraction (yes/no), third grade perineal tear (yes/no), fourth grade perineal tear (yes/no), prelabor erythromycin prescription (yes/no), peripartum antibiotics (defined as having received a new antibiotic prescription in the time period 48 h prior to until 24 h after delivery). PROM was defined using ICD-diagnoses O420, O421, O422 and O429 (*n* = 300, 3.2% in the MPR group, 2.1% in the reference group). Erythromycin is routinely prescribed in cases of preterm premature rupture of membranes prior to 35 weeks of gestation and used as an indirect variable. Multiple gestation (*n* = 102) was not included in the multivariate analyses as none of these women received postpartum antibiotics.

The entire study population and a subgroup of antibiotic-naïve women were analyzed. The subgroup included women not having received antibiotics 48 h prior to until 24 h after delivery. The purpose was to assess the unmitigated effect of MPR on postpartum infection.

Univariate regression analyses assessed risk factors for the prescription of postpartum antibiotics. Significant confounding factors were included in multivariable logistic regression analysis using stepwise backward exclusion by Wald of variables with *P* > 0.10.

Statistical analyses were performed using IBM SPSS Statistics for Macintosh, Version 25.0 (IBM Corporation, Armonk, NY, USA). *C*ategorical variables were analyzed using Chi-squared test or Fisher’s exact test, and continuous variables were analyzed using the Mann–Whitney *U* test. Number needed to harm (NNH) was calculated from rates and adjusted odds ratios (OR) [14]. *P* < 0.05 was regarded as statistically significant.

## Results

Manual placenta removal was performed in 2.5% (*n* = 349) of vaginal births. MPR was significantly associated with increased maternal age, primiparity, induced labor, third and fourth grade perineal tear, an increased use of epidural anesthesia, vacuum extraction, and a longer hospital-stay. Women having undergone MPR lost more blood and blood transfusions were more common.

In the MPR group, 1.7% (*n* = 6) of women were diagnosed with endometritis, compared to 0.2% (*n* = 22) of women with a spontaneous placenta delivery. Endometritis (*P* < 0.001), puerperal fever (*P* < 0.05) and unspecified postpartum infection (*P* < 0.001) were more common in women having undergone MPR.

The variables maternal age, smoking during pregnancy and PROM were not found to be significant confounding factors and thus not included in the multivariate analyses.

As shown in Table 1, MPR was associated with an increased risk of an antibiotic prescription 24 h to seven days postpartum, crude OR = 3.2 (95% CI 2.1–4.7); adjusted OR = 2.9 (95% CI 1.9–4.3).

**Table 1:** Risk factors for prescription of antibiotics, from 24 h postpartum to 7 days postpartum, for women in the entire study population

	Not prescribed antibiotics	Prescribed any type of antibiotic		Univariate		Multivariate*	
	(*n* = 13 487)	(*n* = 390)	*P*	OR (95% CI)	*P*	OR (95% CI)	*P*
Manual removal of placenta	321 (2.4)	28 (7.2)	< 10^–8^	3.2 (2.1–4.7)	< 10^–7^	2.9 (1.9–4.3)	< 10^–6^
BMI (kg/m^2^)	24 (22–27)	24 (22–29)	0.026	1.12 (1.01–1.24)	0.027	1.11 (1.001–1.23)	0.047
Primiparous	5450 (40.4)	216 (55.4)	< 10^–8^	1.83 (1.50–2.24)	< 10^–8^		
Gestational age (weeks + days)	40 + 1 (39 + 3–41 + 0)	40 + 2 (39 + 3–41 + 1)	0.025	1.07 (0.99–1.16)	0.060		
Induction of labor	2119 (15.7)	86 (22.1)	< 10^–3^	1.52 (1.19–1.94)	< 10^–3^		
Epidural	4134 (30.7)	202 (51.8)	< 10^–18^	2.43 (1.99–2.98)	< 10^–17^	1.92 (1.54–2.39)	< 10^–8^
Vacuum extraction	697 (5.2)	62 (15.9)	< 10^–19^	3.5 (2.6–4.6)	< 10^–17^	2.30 (1.69–3.13)	< 10^–6^
Third grade perineal tear	353 (2.6)	35 (9)	< 10^–13^	3.7 (2.6–5.3)	< 10^–11^	1.8 (1.2–2.7)	0.007
Fourth grade perineal tear	3 (0.02)	2 (0.5)	0.007	23.2 (3.9–139.0)	< 10^–3^	7.9 (1.1–56.3)	0.038
Erythromycin	54 (0.4)	4 (1)	0.079	2.6 (0.9–7.2)	0.069		
Peripartum antibiotics^†^	2048 (15.2)	134 (34.4)	< 10^–23^	2.92 (2.36–3.62)	< 10^–22^	2.14 (1.69–2.70)	< 10^–9^

Dichotomous variables, *n* (%); Other variables, median (lower–upper quartile limits)

*BMI* body mass index, *CI* confidence interval, *OR* odds ratio

*Multivariate analysis with backward elimination of insignificant factors by Wald

^†^Received any type of antibiotic 48 h prior to delivery until 24 h postpartum

In the antibiotic-naive subgroup (*n* = 11 695), MPR was associated with an increased risk of antibiotic prescription 24 h to seven days postpartum; crude OR = 2.6 (95% CI 1.5–4.5); adjusted OR = 2.2 (95% CI 1.2–4.0) (Table 2).

**Table 2:** Risk factors for prescription of antibiotics, from 24 h postpartum to 7 days postpartum, for women in the antibiotic-naïve subgroup

	Not prescribed antibiotics	Prescribed any type of antibiotic		Univariate		Multivariate^†^	
	(*n* = 11 439)	(*n* = 256)	*P*	OR (95% CI)	*P*	OR (95% CI)	*P*
Manual removal of placenta	249 (2.2)	14 (5.5)	< 10^–3^	2.6 (1.5–4.5)	< 10^–3^	2.2 (1.2–4.0)	0.007
BMI (kg/m^2^)	24 (22–27)	24 (22–29)	0.025	1.16 (1.02–1.31)	0.023	1.19 (1.05–1.35)	0.006
Primiparous	4275 (37.4)	125 (48.8)	< 10^–3^	1.60 (1.25–2.05)	< 10^–3^		
Gestation age (weeks + days)	40 + 1 (39 + 3–41 + 0)	40 + 3 (39 + 3–41 + 1)	0.055	1.11 (1.01–1.23)	0.039		
Induction of labor	1548 (13.5)	51 (19.9)	0.003	1.59 (1.16–2.17)	0.004		
Epidural anesthesia	3178 (27.8)	112 (43.8)	< 10^–7^	2.02 (1.57–2.60)	< 10^–7^	1.83 (1.40–2.39)	< 10^–5^
Vacuum extraction	526 (4.6)	34 (13.3)	< 10^–9^	3.2 (2.2–4.6)	< 10^–8^	2.6 (1.8–3.9)	< 10^–5^
Third grade perineal tear	168 (1.5)	12 (4.7)	< 10^–3^	3.3 (1.8–6.0)	< 10^–4^	2.3 (1.2–4.4)	0.013
Fourth grade perineal tear	1 (0.009)	0	N/A	N/A	N/A		
Erythromycin	17 (0.1)	2 (0.8)	0.064	5.3 (1.2–23.0)	0.026	6.0 (1.3–27.0)	0.019

Dichotomous variables, *n* (%); Other variables, median (lower–upper quartile limits)

*BMI* body mass index, *N/A* not applicable, *CI* confidence interval, *OR* odds ratio

Women prescribed antibiotics 48 h prior to delivery until 24 h postpartum were excluded

^†^Multivariate analysis with backward elimination of insignificant factors by Wald

Approximately 25% in the MPR group (*n* = 86) were prescribed peripartum antibiotics (48 h prior to until 24 h postpartum). In this group, a greater share of women, who were prescribed peripartum antibiotics, were prescribed antibiotics the first week postpartum as well (16.3%), compared to women not having received peripartum antibiotics (5.3%).

As shown in Table 3, in the antibiotic-naïve subgroup, MPR was associated with an increased risk of prescription of endometritis-antibiotics; crude OR = 3.0 (95% CI 1.7–5.4); adjusted OR = 2.7 (95% CI 1.5–4.9).

**Table 3:** Risk factors for prescription of endometritis-antibiotics, from 24 h postpartum to 7 days postpartum, for women in the antibiotic-naïve subgroup

	Not prescribed endometritis-antibiotics	Prescribed endometritis-antibiotics		Univariate		Multivariate^†^	
	(*n* = 11 490)	(*n* = 205)	*P*	OR (95% CI)	*P*	OR (95% CI)	*P*
Manual removal of placenta	250 (2.2)	13 (6.3)	< 10^–3^	3.0 (1.7–5.4)	< 10^–3^	2.7 (1.5–4.9)	0.001
BMI (kg/m^2^)	24 (22–27)	24 (22–29)	0.037	1.16 (1.01–1.33)	0.037	1.20 (1.04–1.38)	0.011
Primiparous	4299 (37.4)	101 (49.3)	< 10^–3^	1.62 (1.23–2.14)	< 10^–3^		
Gestation age (weeks + days)	40 + 1 (39 + 3–41 + 0)	40 + 3 (39 + 4–41 + 2)	0.040	1.15 (1.02–1.29)	0.020		
Induction of labor	1559 (13.6)	40 (19.5)	0.014	1.54 (1.09–2.19)	0.015		
Epidural anesthesia	3201 (27.9)	89 (43.3)	< 10^–6^	1.99 (1.50–2.62)	< 10^–5^	1.76 (1.31–2.37)	< 10^–3^
Vacuum extraction	532 (4.6)	28 (13.7)	< 10^–8^	3.3 (2.2–4.9)	< 10^–7^	2.7 (1.7–4.1)	< 10^–4^
Third grade perineal tear	170 (1.5)	10 (4.9)	0.001	3.4 (1.8–6.6)	< 10^–3^	2.3 (1.1–4.7)	0.023
Fourth grade perineal tear	1 (0.009)	0	N/A	N/A	N/A		
Erythromycin	19 (0.2)	0	N/A	N/A	N/A		

Dichotomous variables, *n* (%); Other variables, median (lower–upper quartile limits)

*BMI* body mass index, *N/A* not applicable, *CI* confidence interval, *OR* odds ratio

Women prescribed antibiotics 48 h prior to delivery until 24 h postpartum were excluded

^†^Multivariate analysis with backward elimination of insignificant factors by Wald

As presented in Table 4, MPR was associated with a fourfold increased risk (adjusted OR) of being prescribed intravenous antibiotics in the antibiotic-naïve subgroup.

**Table 4:** Risk factors for prescription of intravenous antibiotics, from 24 h postpartum to 7 days postpartum, for women in the antibiotic-naïve subgroup*

	Not prescribed intravenous antibiotics	Prescribed intravenous antibiotics		Univariate		Multivariate^†^	
	(*n* = 11 591)	(*n* = 104)	*P*	OR (95% CI)	*P*	OR (95% CI)	*P*
Manual removal of placenta	253 (2.2)	10 (9.6)	< 10^–3^	4.8 (2.5–9.3)	< 10^–5^	4.0 (2.0–7.9)	< 10^–4^
BMI (kg/m^2^)	24 (22–27)	25 (22–28)	0.084	1.15 (0.95–1.40)	0.16		
Primiparous	4349 (37.5)	51 (49)	0.016	1.60 (1.09–2.36)	0.017		
Gestation age (weeks + days)	40 + 1 (39 + 3–41 + 0)	40 + 2 (39 + 4–41 + 3)	0.099	1.18 (0.99–1.39)	0.053		
Induction of labor	1574 (13.6)	25 (24)	0.002	2.0 (1.3–3.2)	0.002	1.75 (1.10–2.77)	0.018
Epidural anesthesia	3246 (28)	44 (42.3)	0.001	1.9 (1.3–2.8)	0.002	1.59 (1.06–2.38)	0.024
Vacuum extraction	548 (4.7)	12 (11.5)	0.004	2.6 (1.4–4.8)	0.002	1.7 (0.9–3.3)	0.097
Third grade perineal tear	172 (1.5)	8 (7.7)	< 10^–3^	5.5 (2.6–11.6)	< 10^–5^	4.1 (1.9–8.8)	< 10^–3^
Fourth grade perineal tear	1 (0.009)	0	N/A	N/A	N/A		
Erythromycin	19 (0.2)	0	N/A	N/A	N/A		

Dichotomous variables, *n* (%); Other variables, median (lower–upper quartile limits)

*BMI* body mass index, *N/A* not applicable, *CI* confidence interval, *OR* odds ratio

*Women prescribed antibiotics 48 h prior to delivery until 24 h postpartum were excluded

^†^Multivariate analysis with backward elimination of insignificant factors by Wald

A sub-analysis was performed in which total blood loss was adjusted for. In this sub-analysis, MPR was not associated with intravenous antibiotics postpartum, neither in the entire study population, adjusted OR = 1.9 (95% CI 0.9–4.0), nor in the antibiotic-naïve subgroup, adjusted OR = 1.9 (95% CI 0.8–4.5).

In the antibiotic-naïve subgroup, for every 34 women undergoing MPR, one additional woman was prescribed intravenous antibiotics 24 h to seven days postpartum, compared with the reference population (NNH = 34). When calculated based on adjusted OR, NNH was 42.

## Discussion

Our study including 13,877 deliveries showed that MPR was associated with an increased risk of postpartum antibiotic prescription, a proxy variable of infection. The incidence of MPR was 2.5% and MPR was linked with a high-risk pregnancy. This is in line with previous findings [1, 2].

The high frequency of peripartum antibiotics prescribed in the MPR group would be a complicating factor in a randomized controlled trial (RCT). The antibiotic-naïve subgroup represents the women who could gain from prophylactics in conjunction with MPR. In this group, MPR was associated with a fourfold increased risk of being prescribed intravenous antibiotics, suggesting more serious infections.

As many as 25% undergoing MPR received peripartum antibiotics, and it was more common with a postpartum antibiotic prescription among women receiving peripartum antibiotics. This suggests that women receiving peripartum antibiotics were a high-risk group, receiving peripartum antibiotics as well as a new prescription of antibiotics postpartum.

Previous research has defined postpartum endometritis based on documented symptoms and/or ICD-diagnoses [4, 7–9]. However, the extent to which such information is documented in medical records is arbitrary [15]. In our study, more patients were treated for postpartum infection (*n* = 390) than received an ICD-diagnosis of infection (*n* = 289), suggesting that ICD-codes could underestimate the rate of infection. With the aim of performing research based on everyday clinical judgment, we hypothesized that a prescription of antibiotics was a valid end point for infection. A similar design was applied in the ANODE study, an RCT assessing the effect of prophylactic antibiotics after operative vaginal delivery [16].

Difficulties exist in distinguishing different indications for antibiotic treatment near delivery. Underlying reasons could be preexisting infection, prophylaxis (rupture of membranes, colonization of Group β Streptococci, grade three/four perineal tear), or treatment of endometritis which usually develops within 48 h postpartum [17]. Furthermore, evidence suggests that intrauterine infection could be one of the pathophysiological mechanisms leading to placenta retention [18]. Consequently, we performed analyses in a limited time period of 24 h to seven days postpartum and sharpened our end point analyzing endometritis-specific antibiotics and intravenous antibiotics in an antibiotic-naïve subgroup.

If antibiotic prescription is to be a successful tool to measure infection, it is essential that antibiotics are not prescribed in a facile manner. Due to increasing antibiotic resistance, a restrictive policy of antibiotic use has been evolved in Sweden [11]. According to a report by the European Centre for Disease Prevention and Control, the total consumption of antibiotics per capita in Sweden is among the lowest in Europe [19]. This suggests that antibiotic prescriptions can be a functional measure of infection in a Swedish setting.

Blood loss was not included in the multivariate analyses as it is not a confounding variable, being on the pathway between MPR and infection, and a direct effect of the exposure. In a sub-analysis in which blood loss was adjusted for, the risk of intravenous antibiotics after MPR was not significant. Our results support previous studies showing an association between anemia and postpartum infection [7, 8, 20]. Hemorrhage is a possible pathophysiological mechanism linking MPR to postpartum infection.

The WHO recommends prophylactic antibiotics after MPR, although concludes that the level of evidence is low [21]. Swedish guidelines mention the lack of evidence as well [22]. In a review from 2015, Chibueze et al. [23] performed a meta-analysis on three retrospective cohort studies. None of the studies reported any significant difference in the incidence of postpartum endometritis in prophylactic versus reference groups. However, the treatment groups only included 30 to 65 women. The meta-analysis concluded the need for RCTs to elucidate whether patients undergoing MPR would benefit from prophylactic antibiotics.

## Conclusion

Our results support findings that manual placenta removal is associated with an increased risk of postpartum infection [7, 8]. Further studies are needed to determine whether prophylactic antibiotics are warranted to reduce the frequency and gravity of infection.
